# Radial Head Arthroplasty among Patients with Radial Head Fracture in a Tertiary Care Centre: A Descriptive Cross-sectional Study

**DOI:** 10.31729/jnma.7828

**Published:** 2022-10-31

**Authors:** Manoj Kandel, Deepak Banjade, Shrawan Kumar Thapa, Prakash Kandel, Bishwa Raj Adhikari, Kabin Neupane

**Affiliations:** 1 Department of Orthopaedics, Bharatpur Hospital, Bharatpur, Chitwan, Nepal

**Keywords:** *arthroplasty*, *elbow prosthesis*, *radius fractures*

## Abstract

**Introduction:**

Proximal radial fractures are a common type of fracture around the elbow joint. Among these comminuted radial head fractures are commonly associated with secondary injuries and instability of the elbow joint. Management of the radial head in such cases is very important in restoring the stability of the elbow joint and starting early mobilization. The aim of this study was to find out the prevalence of radial head arthroplasty among patients with radial head fracture in a tertiary care centre.

**Methods:**

A descriptive cross-sectional study was conducted from 25 January 2019 to 25 December 2020 among patients with radial head fracture at the Department of Orthopaedics of a tertiary care centre. Ethical approval was obtained from the Institutional Review Committee (Reference number: 076/77-08A). A convenience sampling method was used. The study group consisted of patients between 20 to 60 years of age with isolated radial head fractures. Radial head arthroplasty was done for Mason types III and IV fractures and functional outcome was calculated postoperatively with Mayo elbow score on follow-up at 3-, 6- and 12-month intervals. Point estimate and 95% Confidence Interval were calculated.

**Results:**

Out of 96 patients with radial head fracture, 22 (22.92%) (17.59-28.25, 95% Confidence Interval) underwent radial head arthroplasty. The mean Mayo elbow score was 96.33±7.74 at 12 months of follow-up.

**Conclusions:**

The prevalence of radial head arthroplasty among irreparable radial head fractures was similar compared to other studies done in a similar setting.

## INTRODUCTION

Radial head fractures constitute one-third of all elbow injuries and 2-5% of all adult fractures.^1^ Injury to collaterals of the elbow results in instability of the joint in these fractures. The primary stabilizer of the elbow in these cases is the radial head and is important for early mobilization.^2^ Mason I and II injuries are amenable to conservative methods or surgical fixation if displaced. Mason III and above include comminuted fractures that are not reconstructible, options being radial head excision with or without replacement.^3^

After radial arthroplasty, stability and kinematics of the elbow are similar to the anatomical radial head.^4^ Studies have shown altered stability and kinematics of the elbow after excision alone without replacement.

Radial arthroplasty offers better results than radial head excision alone.^5^ This study analyses the functional outcome after treatment of radial head type III with metallic radial head prostheses with help of the Mayo elbow score.

The objective of this study was to find out the prevalence of radial head arthroplasty among patients with radial head fracture in a tertiary care centre.

## METHODS

This descriptive cross-sectional study was conducted from 25 January 2019 to 25 December 2020 in the Department of Orthopaedics of Bharatpur Hospital. Ethical approval was obtained from the Institutional Review Committee (IRC) of the same institute (Reference number: 076/77-08A). The study group consisted of patients between 20 to 60 years of age with isolated radial head fractures. Patients who had compound fractures and pathological fractures due to malignancy were excluded. A convenience sampling method was used. The sample size was calculated by using the following formula:


$$\begin{array}{ll} \text{n} & ={\text{Z}}^{2}\times \frac{\text{p}\times \text{q}}{{\text{e}}^{\text{2}}}\\ & =1.96^{2}\times \frac{0.50\times 0.50}{0.10^{2}}\\ & =96 \end{array}$$

Where,

n= minimum required sample size
Z= 1.96 at 95% Confidence Interval (CI)
p= prevalence of taken as 50% for maximum sample size calculation
q= 1-p
e= margin of error, 10%

The calculated sample size was 96. After managing the patients in the casualty department with advanced trauma life support and ruling out other life-threatening injuries, those with radial head fractures were evaluated with an X-ray of the elbow-anteroposterior and lateral views and when required, additional oblique views, Computed Tomography (CT) scans, and Magnetic Resonance Imaging (MRI) were taken to characterise the fracture and associated injuries. The fractures were immobilised in the above elbow posterior slab. The patients were explained in detail the pros and cons of the procedure and consent was taken from all patients who were recruited for this procedure.

Standard Kocher's approach was used for surgery with tourniquet application. The patients with Mason type III and above were treated with titanium radial head prostheses. After radial head replacement with a prosthesis, the annular ligaments were repaired. Prophylaxis against heterotopic ossification was done with 3 weeks course of indomethacin. Active and passive physiotherapy started on the fourth postoperative day. All patients were followed up clinically and evaluated radiologically for a mean of 12 months. The clinical evaluation was performed using the Mayo elbow performance score (MEPS). The assessment included a record of the patient's pain level, range of movement at the elbow, elbow stability, and functional level. Each patient's affected range of movement was compared with the contralateral elbow. The MEPS results were classified as excellent (≥90), good (75-89), fair (60-74), or poor (<60).^6,7^

The details of each patient and the variable were entered in Microsoft Excel 2016 and data analysis was done using IBM SPSS Statistics 25.0. Point estimate and 95% CI were calculated.

## RESULTS

Out of 96 patients with radial head fractures, arthroplasty was performed in 22 (22.92%) (17.5928.25, 95% CI). The mean age of the patient was 38±10.92 years with a range of 21 to 52 years. The mode of injury in 12 (54.55%) of them was a fall on an outstretched hand, 6 (27.27%) had a road traffic accident, and 4 (18.18%) were followed by an assault. The radial head replacement was done in all patients as a primary procedure. Males and females were 16 (72.73%) and 6 (27.27%) respectively. In our study, 4 (18.18%) patients had lateral collateral ligament (LCL) injuries (Table 1).

**Table 1:** Co-existing injuries and events (n = 22).

Co-existing injuries around elbow	n (%)
MCL injury	8 (36.36)
LCL injury	4 (18.18)
Elbow dislocation+LCL injury+MCL injury	3 (13.63)
Not associated with injury	7 (31.18)

**Figure 1:**
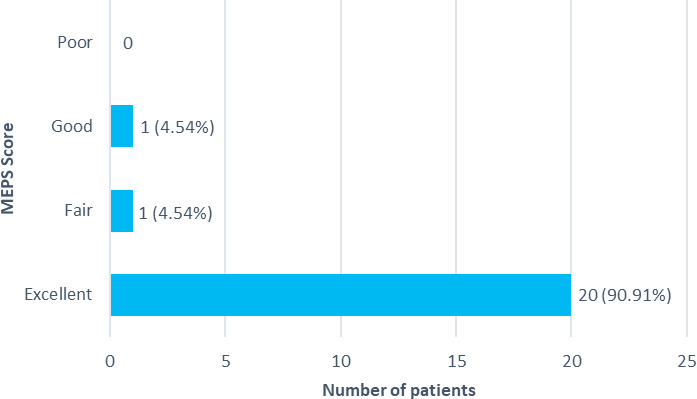
Total Mayo elbow performance score at the end of 12-month follow-up (n= 22).

A total of 2 (9.09%) patients were comorbid with diabetes mellitus and hypertension, 4 (18.18%) patients with hypertension only, and 1 (4.54%) patient with rheumatoid arthritis. Patients with smokers were 12 (54.54%). Among patients with radial head arthroplasty 20 (90.91%) patients showed excellent elbow performance scores, 1 (4.54%) of them showed good and 1 (4.54%) showed fair result. Poor result as per MEPS scoring was seen in none (Figure 1).

At the end of 12 months, the operated extremity has mean supination of 85.92±1.41°, pronation of 83.73±1.70°, and mean flexion arc of 115.91 ±9.20°. Signs of infection, implant loosening or elbow instability was seen in 0 (0%) (Table 2).

**Table 2:** Subjective status of the radial head arthroplasty at the end of 12 months follow-up (n= 22).

Clinical	Mean±SD	Minimum	Maximum
Flexion	115.91±9.206	90	124
Supination	85.91±1.411	84	88
Pronation	83.73±1.778	80	88
Pain	43.64±4.414	30	45
ROM	19.55±1.471	15	20
Stability	10	10	10
ADL	24.32±2.338	15	25
Total MEPS	96.36±7.743	70	100

Out of 22 patients, 3 (13.64%) patients had superficial wound infectionswhich healed afterwounddebridement and repeated dressing and 1 (4.54%) patient had elbow joint stiffness at first-month of follow-up but which improved later to functional range of movement.

## DISCUSSION

The prevalence of arthroplasty cases among radial head fractures was 22.92%. In the literature review, up to 33.34% of patients get replacement arthroplasty for radial head fractures, which is higher compared to our study.^8^

Historically, the radial head was believed to be expendable. Now it is universally appreciated as a vital elbow structure for forearm and elbow stability. Radial head resection overloads the coronoid process, the elbow then depends on the MCL to prevent valgus deformity.^9^ If the interosseous membrane is disrupted the radius is proximally migrated and for each mm of radial shortening, the distal ulnar load increases by approximately 10%. Comminuted fractures of the radial head where internal fixation is not possible and if no other lesion affecting the stability of the elbow simple excision is a good option.^10^ In the long-term cases of inappropriate excision of radial head surgery may accelerate the onset of osteoarthritis.^11^ Therefore, radial head arthroplasty is indicated if there are instabilities in the elbow. For the first time in 1941, speed proposed an acrylic prosthetic replacement of the radial head. Later on, this was discarded due to fractures of the prosthesis itself. Silastic prosthesis was introduced in the 1970s which act as a spacer only without giving any biomechanical advantage in weight transmission and also caused synovitis. Literature supports metallic radial head replacement as it restores the axial stiffness of the forearm to normal whereas excision allows abnormal proximal migration, especially under load.^12^

In one study, surgical fixation for Mason type III fractures having more than three articular fragments were found more likely to result in unsatisfactory outcomes. Fragmentation and instability are the characteristics that seem to affect the results of internal fixation. The presence of more than three fragments in an unstable displaced fracture portends poor results with salvage procedure. They concluded that these fractures should be best treated with replacement arthroplasty.^13^ In a retrospective study of 10 patients with Mason type III fractures treated with radial head replacements, the average age was 47 years. They reported both assessments comparing the function of the injured arm with the contralateral arm. Average pronation was 78° compared to 79°, supination compared to 77°, elbow flexion 140° compared to 143°, and elbow extension deficit was 8° compared to full extension on the uninjured side.^14^ In our study, average flexion was 125°, average extension deficit was 7°, average pronation was 83°, and average supination was 85°. Similar to our study, there were a few complications like elbow stiffness, varying levels of heterotopic ossification, one superficial infection and one patient developed a regional sympathetic-mediated pain syndrome in six patients but they improved over a period of time after wound care and physiotherapy.

A study in China compared radial head replacement and open reduction internal fixation for comminuted unstable radial head fractures. Two years of follow-up showed patients with radial head replacement had significantly better range of motion and lesser complications.^8^ Similar result was seen in a study done by Belgian surgeons. In their study out of 11 patients with the mean follow-up of 32 months, they found four cases with excellent results, four cases with good results, two cases with fair results, and one had poor result which is comparable with our results.^15^

A retrospectively reviewed study of 17 patients with an average follow-up of 106 months (range 78-139 months) where an acute radial head replacement was performed in nine patients and delayed replacement was performed in eight patients with heterogeneous injury patterns showed that despite the complex injury patterns, the average MEPS score was 90.8, with 16 (94%) good or excellent results.^16^ In another study with 16 patients treated with a titanium radial head prosthesis, the average MEPS score at final follow-up (average 2.8 years) was 87 (range 65-100) with 13 (81%) obtaining good or excellent results. The average VAS score was 1.7 (range 0-4.5) and seven of nine employed patients were able to return to work which is comparable with our results.^17^ One of the first series using titanium radial head prostheses was published in 1981. They reported good or excellent results in 14 (93%) of 15 patients at an average follow-up of 6.9 years.^18^ A total of 12 patients were reviewed in a French hospital with a mean follow-up of 49 months where an acute radial head replacement was performed in five patients and delayed replacement in seven patients after failed excision. All patients who underwent acute reconstruction had good results, with an average Mayo Elbow Performance Score (MEPS) of 84.^19^ In another study consisting of 55 patients with non-reconstructible radial head fractures treated acutely with a smooth-stemmed modular metallic radial head implant were retrospectively reviewed. At a mean of 8.2±2.9 years, the mean arc of flexion (and standard deviation) of the affected elbow was 11±14° to 137±15°. The mean Mayo Elbow Performance Index (MEPI) was 91 ±13 points.^20^ These mid-term outcomes of radial head arthroplasty are comparable with previously reported short-term outcomes, with no evidence of functional deterioration. A comparative study done between radial head resection and open reduction internal fixation shows that fixation had better outcomes than resection.^21^ While two randomized studies were performed in an attempt to provide evidence comparing open reduction internal fixation and radial head replacement. Both of these studies provide data to suggest that radial head replacement provides a better functional outcome than internal fixation.^22^ This shows radial head replacement is the best of the three procedures to preserve the normal functional anatomy of the elbow. With magnetic resonance imaging (MRI) in the setting of radial head fracture, studies found even higher incidences of concomitant ligamentous injury than with physical or clinical examination alone. A total of 92% incidence of associated injuries in Mason type II and type III radial head fractures were diagnosed.^23^ In this respect, the medial collateral ligament is the primary constrainer in valgus stress. The radial head contributes secondarily to valgus stability and its preservation is mandatory in case of fractures that involve soft tissue and ligaments to avoid chronic instability. A similar study reported that 100% of Mason type III radial head fractures had associated injuries.^24^ This might suggest that radial head arthroplasty is indicated for all non-reconstructable radial head fractures whether isolated or not.

This study was based on a single centre and the data was collected using convenience sampling. So, this might not be generalizable to other hospitals.

## CONCLUSIONS

The prevalence of radial head arthroplasty among radial head fractures is lower as compared to other studies done in similar settings. The lack of implant availability and surgical skill could have an impact on this lesser prevalence. A good operative technique, choice of implant, and early postoperative mobilization could improve the final functional outcome.
